# Perceived burden and coping strategies among caregivers of mentally ill patients

**DOI:** 10.6026/973206300220880

**Published:** 2026-02-28

**Authors:** Mahida Sadiya Zakirhusen, N. Siva Subramanian, Mahalakshmi B

**Affiliations:** 1 Department of Psychiatric Nursing, Nootan College of Nursing, Sankalchand Patel University, Visnagar, Gujarat, India; 2 Department of Paediatric Nursing, Nootan College of Nursing, Sankalchand Patel University, Visnagar, Gujarat, India

**Keywords:** Caregiver burden, coping strategies, psychological well-being, mental illness, family caregivers

## Abstract

The caregivers of mentally ill patients are facing a lot of burden, Strain, unhealthy life style and not able to cope with their mentally ill patient and needs support for the family. Therefore, it is of interest to assess perceived burden, coping strategies and psychological well-being among caregivers of mentally ill patients in selected psychiatric hospitals of Gujarat. Hence, a total of 100 caregivers were selected using purposive sampling. The findings showed that 63% of caregivers experienced high burden, while 37% reported very high burden. Nearly 75% of caregivers had inadequate coping strategies. Data shows the substantial caregiver burden and the need for targeted interventions to improve coping and psychological well-being. This study advances knowledge by documenting high caregiver burden, inadequate coping and compromised psychological well-being among caregivers of mentally ill patients in Gujarat. Thus, demographic associations, supporting the need for structured psychosocial and nursing interventions.

## Background:

Family caregivers of individuals with mental illness play a critical role in patient care, often serving as primary support providers in community and home settings. However, caregiving for mentally ill patients is associated with substantial physical, emotional, financial and social burden, commonly referred to as caregiver burden [1]. This burden encompasses objective dimensions (practical demands of care) and subjective dimensions (emotional strain and perceived stress), both of which significantly impact caregivers' quality of life and mental health [2]. Caregiver burden in mental illness is particularly pronounced due to the chronic and relapsing nature of psychiatric disorders, unpredictable behavioral symptoms, stigma and prolonged duration of caregiving [3]. Studies have documented high prevalence of burden among caregivers of patients with schizophrenia, bipolar disorder, depression and other severe mental illnesses, with rates ranging from 40% to 90% across different populations [4]. In India, where family-based care remains the predominant model due to limited institutional resources, cultural expectations and economic constraints, caregivers often face additional challenges including inadequate professional support, lack of respite services and financial strain [5]. Coping strategies-the cognitive and behavioral efforts employed to manage stressful situations-are crucial determinants of caregivers' adaptation and resilience [6]. Effective coping mechanisms such as problem-solving, seeking social support and positive reframing are associated with reduced burden and better psychological outcomes, whereas maladaptive strategies like avoidance, denial and self-blame tend to exacerbate distress [7]. Psychological well-being- encompassing emotional balance, life satisfaction, positive affect and sense of purpose-is frequently compromised among caregivers due to chronic stress exposure [8].

Diminished psychological well-being not only affects caregivers' health and functioning but may also impair their capacity to provide effective care, potentially worsening patient outcomes [9]. Understanding the relationship between caregiver burden, coping strategies and psychological well-being is therefore essential for developing comprehensive support interventions. Despite growing recognition of caregiver burden globally, empirical research from Gujarat and similar Indian contexts remains limited, particularly regarding the interrelationships among burden, coping and well-being [10]. Moreover, examination of demographic factors that may influence these variables-such as caregiver age, gender, education, income, occupation and duration of caregiving-can inform targeted intervention strategies [11]. Therefore, it is of interest to assess the levels of perceived burden, coping strategies and psychological well-being among caregivers of mentally ill patients at selected psychiatric hospitals in Gujarat and to examine correlations among these variables and their associations with demographic characteristics.

## Methodology:

## Research approach and design:

A quantitative approach with cross-sectional descriptive design was employed to assess perceived burden, coping strategies and psychological well-being among caregivers and to examine correlations and demographic associations.

## Setting and population:

The study was conducted at selected psychiatric hospitals in Gujarat, India. The target population comprised primary family caregivers of patients diagnosed with mental illness who were receiving treatment at these facilities.

## Sample and sampling technique:

A total of 100 caregivers were recruited using non-probability purposive sampling based on predefined inclusion and exclusion criteria.

## Data collection tools:

Data were collected using structured, validated instruments: Socio-demographic Proforma to gather demographic data, Perceived Burden Scale to assessing caregiver burden across multiple domains and coping Strategies Inventory to measuring adaptive and maladaptive coping mechanisms. Psychological Well-Being Scale: A standardized measure assessing emotional balance, life satisfaction, positive affect and purpose. Categories include excellent well-being, good well-being and fair well-being and need attention.

## Data analysis:

Data were analyzed using SPSS version 20.0. Descriptive statistics (frequency, percentage, mean, standard deviation) were used to summarize demographic characteristics and levels of burden, coping and well-being. Spearman's rank correlation coefficient was calculated to examine relationships between burden-coping and burden-well-being. Chi-square tests assessed associations between outcome variables and demographic characteristics. Statistical significance was set at p<0.05.

## Results:

Table 1 show that most caregivers were middle-aged 50 (50%), male 68 (68%) and married 80 (80%). The majority had moderate to higher education, earned above Rs. 5,000 per month 65 (65%), were engaged in physically demanding work 48 (48%) and had provided care for more than one year 70 (70%), indicating sustained caregiving involvement. Table 2 show all demographic variables showed non-significant associations with perceived burden, coping strategies and psychological well-being, as all p-values exceeded 0.05 in the chi-square tests. This indicates that caregiver burden, coping deficits and compromised well-being were uniformly distributed across age, gender, marital status, education, income, occupation and duration of caregiving. The absence of significant demographic effects suggests that these challenges are pervasive and not confined to any specific subgroup of caregivers. Figure 1 (see PDF) show pperceived burden was entirely high to very high, with high 63 (63%) and very high 37 (37%). Coping was mostly inadequate (Inadequate 75 (75%), moderately adequate 25 (25%)) and psychological well-being was largely fair (Good 14 (14%), Fair 70 (70%), Need attention 16 (16%)).

## Discussion:

This cross-sectional study revealed alarmingly high levels of perceived burden among caregivers of mentally ill patients, with 100% of the sample experiencing high or very high burden. A study by Lauber *et al.* [11] found that caregivers of patients with exacerbating schizophrenia experienced substantial burden with majority reporting high levels, which is consistent with our results. Research by Awad and Voruganti [12] documented that caregiver burden in schizophrenia is pervasive and affects multiple life domains including emotional, physical and financial aspects, which is consistent with our results. The universal presence of high burden in this sample underscores the severe strain imposed by caregiving responsibilities in psychiatric illness. The chronic nature of mental disorders, unpredictable symptom exacerbations and behavioral disturbances contribute significantly to caregiver burden. A study by Schene *et al.* [13] reported that family caregiving in schizophrenia involves multiple domains of distress including emotional burden and disruption of daily activities, which is consistent with our results. Additionally, Gonçalves-Pereira *et al.* [14] found in a cross-cultural study that psychosis had significant impact on Portuguese caregivers with high burden levels across cultural contexts, which is consistent with our results. Social stigma and limited professional support systems in this setting likely amplify the burden experienced by caregivers. Research by Saunders [15] demonstrated that families living with severe mental illness face substantial challenges due to inadequate community resources and social isolation, which is consistent with our results. The predominance of inadequate coping strategies (75%) in this sample is particularly concerning. Effective coping mechanisms are essential protective factors that mediate the relationship between stressors and psychological outcomes. A study by Folkman and Moskowitz [16] established that coping strategies significantly influence stress outcomes and psychological adaptation, which is consistent with our results. The deficit in adaptive coping observed here suggests that caregivers lack adequate knowledge, skills, or resources to effectively manage caregiving demands. Research by Szmukler *et al.* [17] found that caregivers of patients with serious mental illness often lack effective coping resources leading to increased burden, which is consistent with our results. This may be attributable to insufficient psychoeducation and absence of caregiver training programs. A study by Martens and Addington [18] demonstrated that psychological well-being of family members of individuals with schizophrenia is compromised due to lack of adequate support and training, which is consistent with our results. Limited access to mental health professionals and social isolation further compound the problem. Research by Pai and Kapur [19] showed that family burden is exacerbated by inadequate professional support systems in community settings, which is consistent with our results. Cultural factors that discourage help-seeking behaviors may also play a role. A study by Tanna *et al.* [20] validated the burden assessment schedule in Indian context and found that cultural factors influence how caregivers perceive and cope with burden, which is consistent with our results. Our finding of a significant negative correlation between perceived burden and psychological well-being (r=-0.237, p=0.018) confirms the adverse mental health impact of sustained caregiving strain.

A study by van Wijngaarden *et al.* [21] found significant negative associations between caregiver burden and quality of life in schizophrenia caregivers across European populations, which is consistent with our results. This relationship has been consistently documented in the literature. Research by Veltman *et al.* [22] demonstrated that providing care to relatives with chronic mental illness negatively impacts caregivers' psychological health and well-being, which is consistent with our results. Meta-analyses have shown that caregiver burden is a strong predictor of depression, anxiety and diminished quality of life. A comprehensive meta-analysis by Pinquart and Sörensen [23] found that caregiver burden was associated with increased depression and reduced well-being across diverse caregiving contexts, which is consistent with our results. The direction and significance of this correlation underscore the urgent need for interventions that address both burden reduction and psychological support for caregivers. A study by Gaugler *et al.* [24] showed that reducing caregiver burden leads to improvements in depression and overall mental health outcomes, which is consistent with our results. Interestingly, we found no significant correlation between perceived burden and coping strategies (r=0.084, p=0.408). This unexpected finding contrasts with theoretical models suggesting that effective coping moderates the impact of stressors on outcomes. Research by Rui & Guo [25] established the stress-buffering hypothesis showing that coping resources typically moderate stress outcomes, which differs from our findings. Several explanations are possible: First, the restricted range of coping scores (with 75% in the inadequate category) may have limited statistical power to detect associations. The research study by Thakur *et al.* [26] revealed that Caregivers of patients with major mental illness experience substantial burdens. Appropriate interventions could improve coping strategies and reduce the burden of caregivers.

## Conclusion:

We show that caregivers of mentally ill patients in Gujarat experience uniformly high burden with inadequate coping and poor psychological well-being. The significant negative correlation between burden and well-being confirms the harmful mental health effects of prolonged caregiving. The absence of demographic predictors indicates that caregiver challenges are widespread, highlighting the need for universal support interventions.

## Figures and Tables

**Table 1:** Frequency and percentage distribution of demographic characteristics of caregivers (N=100)

**Demographic Variable**	**Category**	**Frequency (f)**	**Percentage (%)**
Age	18-38 years	18	18
	38-58 years	50	50
	58 and above	32	32
Gender	Male	68	68
	Female	32	32
Marital Status	Married	80	80
	Unmarried	20	20
Educational Status	Elementary	27	27
	Higher secondary	42	42
	Graduate	31	31
Monthly Income	Less than Rs. 3,000	11	11
	Rs. 3,000-5,000	24	24
	More than Rs. 5,000	65	65
Occupation	Sedentary	15	15
	Moderate work	36	36
	Heavy worker	48	48
	None	1	1
Duration of Caregiving	Less than one year	30	30
	More than one year	70	70

**Table 2:** Association between demographic variables and study outcomes: chi-square analysis (N=100)

**Demographic Variable**	**Perceived Burden χ^2^ (df)**	**Perceived Burden p-value**	**Coping Strategies χ^2^ (df)**	**Coping Strategies p-value**	**Psychological Well-Being χ^2^ (df)**	**Psychological Well-Being p-value**
Age	4.577 (2)	0.101	2.259 (2)	0.323	0.844 (4)	0.933
Gender	0.920 (1)	0.338	0.000 (1)	1	3.230 (2)	0.199
Marital Status	3.475 (1)	0.062	1.333 (1)	0.248	1.205 (2)	0.547
Educational Status	0.566 (2)	0.753	0.494 (2)	0.781	2.615 (4)	0.624
Monthly Income	0.299 (2)	0.861	3.543 (2)	0.17	5.958 (4)	0.202
Occupation	4.684 (3)	0.196	3.578 (3)	0.311	7.119 (6)	0.31
Duration of Caregiving	0.002 (1)	0.964	0.571 (1)	0.45	0.570 (2)	0.752
Note: All p-values >0.05
indicating no significant associations;
df = degrees of freedom.
